# Magnetic field detection limits for ultraclean graphene Hall sensors

**DOI:** 10.1038/s41467-020-18007-5

**Published:** 2020-08-20

**Authors:** Brian T. Schaefer, Lei Wang, Alexander Jarjour, Kenji Watanabe, Takashi Taniguchi, Paul L. McEuen, Katja C. Nowack

**Affiliations:** 1 Laboratory of Atomic and Solid State Physics, Cornell University, Ithaca, NY 14853 USA; 2 Kavli Institute at Cornell for Nanoscale Science, Cornell University, Ithaca, NY 14853 USA; 3 National Institute for Materials Science, 1-1 Namiki, Tsukuba, 305-0044 Japan

**Keywords:** Electronic properties and devices, Sensors, Electronic and spintronic devices

## Abstract

Solid-state magnetic field sensors are important for applications in commercial electronics and fundamental materials research. Most magnetic field sensors function in a limited range of temperature and magnetic field, but Hall sensors in principle operate over a broad range of these conditions. Here, we evaluate ultraclean graphene as a material platform for high-performance Hall sensors. We fabricate micrometer-scale devices from graphene encapsulated with hexagonal boron nitride and few-layer graphite. We optimize the magnetic field detection limit under different conditions. At 1 kHz for a 1 μm device, we estimate a detection limit of 700 nT Hz^−1/2^ at room temperature, 80 nT Hz^−1/2^ at 4.2 K, and 3 μT Hz^−1/2^ in 3 T background field at 4.2 K. Our devices perform similarly to the best Hall sensors reported in the literature at room temperature, outperform other Hall sensors at 4.2 K, and demonstrate high performance in a few-Tesla magnetic field at which the sensors exhibit the quantum Hall effect.

## Abstract (web summary)

The development of high-performance magnetic field sensors is important for magnetic sensing and imaging. Here, the authors fabricate Hall sensors from graphene encapsulated in hBN and few-layer graphite, demonstrating high performance over a wide range of temperature and background magnetic field.

## Introduction

Hall-effect sensors are attractive for a variety of magnetic field sensing applications ranging from position detection in robotics^1,2^ and tracking nanoparticles in biological systems^3^ to fundamental studies of magnetism^4^ and superconductivity^5–7^. The sensitive area of a Hall sensor consists of a material with low charge carrier density patterned into a cross shape. Current flowing along the length of the cross produces a transverse voltage that is directly proportional to the magnetic field perpendicular to the Hall cross. Because of this straightforward measurement scheme, Hall sensors provide an accessible means of performing noninvasive measurements of magnetic fields. This operating principle suggests sensitivity over a broad range of temperatures and magnetic fields, whereas other types of sensors, including SQUID magnetometers^5,8,9^ and magnetoresistive sensors^2,10^, only remain sensitive at cryogenic temperatures or small magnetic fields. Hall sensors with a micrometer-scale sensitive area are well-suited for probing mesoscopic magnetic and superconducting structures and devices, with the sensor interfaced directly with the structure^3,4,7^ or integrated into a scanning probe microscope^5,6,11–15^. In scanning Hall probe microscopy, the spatial resolution of measurements is limited by a combination of scan height and sensor size, suggesting development of well-performing sensors with small sensitive areas^5,9,11–13^.

In an ideal Hall-effect sensor, the deflection of electric current in an out-of-plane magnetic field *B* produces a transverse (Hall) voltage response *V*_H_ = *BI*/(*n*e) = *IR*_H_*B*, where *I* is the bias current, *n* is the two-dimensional charge carrier density, e is the electron charge, and *R*_H_ = *I*^−1^(∂*V*_H_/∂*B*) is the Hall coefficient. This voltage response and the Hall voltage noise *S*_*V*_^1/2^ combined give the magnetic field detection limit *S*_*B*_^1/2^ = *S*_*V*_^1/2^/(*IR*_H_). The quantity *S*_*B*_^1/2^ multiplied by the square root of the measurement bandwidth gives the smallest detectable change in magnetic field. Thermal Johnson noise, which is independent of frequency and proportional to the square root of the device resistance, provides a fundamental lower bound for the voltage noise. The desire to minimize Johnson noise suggests that the ideal material system for Hall sensors combines low carrier density for a large Hall coefficient and high carrier mobility for a low device resistance^11^. However, contributions from flicker (“1/*f*”) noise and random telegraph noise often dominate the total noise at practically relevant kHz frequencies, requiring individual characterization of each material system^11,13,16,17^.

Ultraclean graphene is a promising material system for high-performing Hall sensors. Whereas carrier mobility decreases at low carrier density in most semiconductor-based two-dimensional electron systems^18^, in graphene the mobility is enhanced at low carrier density in the absence of long-range impurity scattering^19^. Encapsulation in hexagonal boron nitride (hBN) enables access of this low-density, high-mobility regime^20^. Indeed, hBN-encapsulated graphene is a promising material platform for Hall sensors with low 1/*f* noise in micrometer-scale devices^21^ leading to low magnetic field detection limits^17^ at room temperature. Recent work suggests that using few-layer graphite (FLG) as a gate electrode in addition to hBN encapsulation yields ultraclean graphene devices with exceptional electronic quality^22–24^.

Here, we fabricate Hall sensors from hBN-encapsulated monolayer graphene (MLG) with either Ti/Au metal or FLG gate electrodes. Tuning the carrier density via electrostatic gating enables optimization of the magnetic field detection limit under different operating conditions. We obtain performance comparable to that of high-performing micrometer-scale Hall sensors reported in the literature at room temperature, demonstrate the best reported performance at low temperature, and operate in a high background magnetic field at which the sensors exhibit the quantum Hall effect.

## Results

### Detection limits for micrometer-scale Hall sensors

**Fig. 1:**
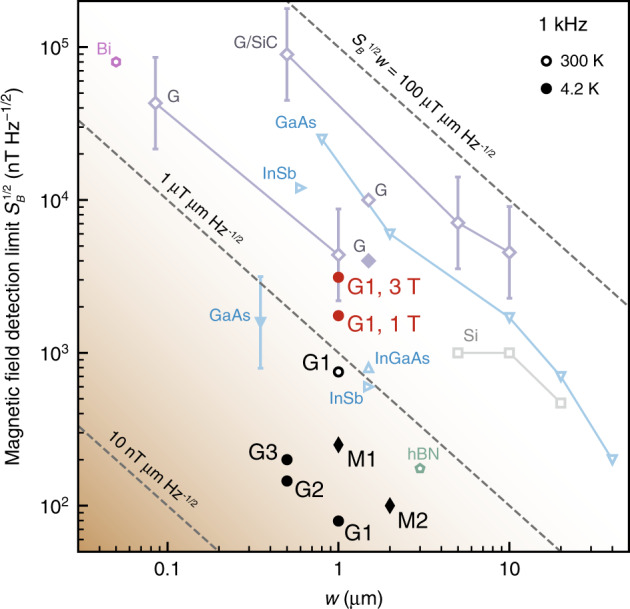
Performance of micrometer-scale Hall sensors. Minimum magnetic field detection limit *S*_*B*_^1/2^ at 1 kHz vs. the width *w* of Hall sensors reported here and in the literature. The black markers show the best performance of our graphite-gated (circles; G1–G3) and metal-gated (diamonds; M1 and M2) devices in zero background magnetic field, and the red circles show the performance of G1 in 1 T and 3 T background field as indicated. All other markers are estimates of the best performance in zero background field of devices made from semiconductor- and graphene-based structures, including graphene grown by chemical vapor deposition (“G”), epitaxial graphene (“G/SiC”), and hBN-encapsulated exfoliated graphene (“hBN”). Filled (open) markers correspond to measurements at 4.2 K (300 K). Solid lines are a guide to the eye connecting markers corresponding to the same material and fabrication process. Dashed lines mark constant *S*_*B*_^1/2^*w*. Markers with error bars are extrapolated from measurements reported at different frequencies, assuming the noise is dominated by 1/*f* noise and scales as *f*^−*α*^ (error bars mark the range 0.4 < *α* < 0.6).

Figure 1 summarizes our main result. We compare the minimum magnetic field detection limit *S*_*B*_^1/2^ for our devices (black markers) with corresponding measurements for high-performing micrometer-scale Hall sensors reported in the literature (see Supplementary Table 1)^3,11–15,17,25–27^. We choose a reference frequency of 1 kHz at which 1/*f* noise is the dominant noise component (see below). The amplitude of 1/*f* noise varies across devices, depending on the material system and external factors including fabrication processing history, choice of substrate, dielectric environment, types of contacts, and biasing conditions^16^. Despite the wide variety of mechanisms causing 1/*f* noise, there are some commonly observed dependencies. Typically, the amplitude of the 1/*f* noise power spectral density increases for smaller devices as 1/*A*, where *A* is the device area. The magnetic field detection limit depends on the square root of the Hall voltage power spectral density, in turn suggesting an approximate scaling of the detection limit *S*_*B*_^1/2^ ∝ *A*^−1/2^ ∝ *w*^−1^ with device size *w* for Hall sensors^5,13^. Therefore, the metric *S*_*B*_^1/2^*w* is typically used to evaluate the performance of Hall sensors across materials and device sizes^17^.

According to this metric, devices with similar performance lie along the dashed diagonal lines of constant *S*_*B*_^1/2^*w* in Fig. 1, with the best-performing devices located towards the lower left corner. At room temperature, the performance of device G1, a graphene Hall sensor with FLG gates, is similar to that of the best sensors made from InGaAs^26^, InSb^15^, and hBN-encapsulated graphene^17^. At low temperature (4.2 K), the detection limit of device G1 decreases by an order of magnitude, and we obtain the smallest value of *S*_*B*_^1/2^*w* reported for any Hall sensor to date. Additional graphite-gated devices (G2 and G3) show performance consistent with an approximate *w*^−1^ scaling of the detection limit. However, hBN-encapsulated graphene devices with metal gates (M1 and M2) exhibit larger detection limits than graphite-gated devices at low temperature (see Supplementary Table 2). At room temperature, device G1 performs similarly to hBN-encapsulated devices without graphite gates (labeled “hBN”) reported previously^17^. This is consistent with the observation that graphite gates improve the electronic properties of graphene predominantly at low temperature. Specifically, graphite gates reduce the intrinsic charge inhomogeneity in graphene devices^22–24^, making mobile carrier densities as low as ~2 × 10^9^ cm^−2^ accessible and leading in turn to a larger attainable Hall coefficient. However, at room temperature thermal excitation of charge carriers and acoustic phonon scattering increase the charge inhomogeneity and limit the carrier mobility^20,28,29^.

We furthermore demonstrate a small detection limit even in several Tesla background magnetic field. Hall sensors based on high-mobility two-dimensional conductors are not typically compatible with high background magnetic fields because these sensors exhibit the quantum Hall effect (QHE). The QHE creates wide regions of parameter space in which the Hall voltage is constant either as a function of magnetic field or carrier density. Here, we take advantage of electrostatic gating to tune the carrier density to a value at which the Hall voltage changes with magnetic field. In this way, we achieve a low magnetic field detection limit at high background magnetic field despite the presence of the QHE. At low temperature and large background magnetic field, device G1 maintains a detection limit of ~2–3 μT Hz^−1/2^ at 1 kHz. The detection limit is larger compared to that measured at zero background magnetic field both due to an increase in voltage noise and a reduction in the Hall coefficient (see below). Nevertheless, the detection limit still remains comparable to that of many high-performing Hall sensors tested at zero magnetic field.

### Device structure

**Fig. 2:**
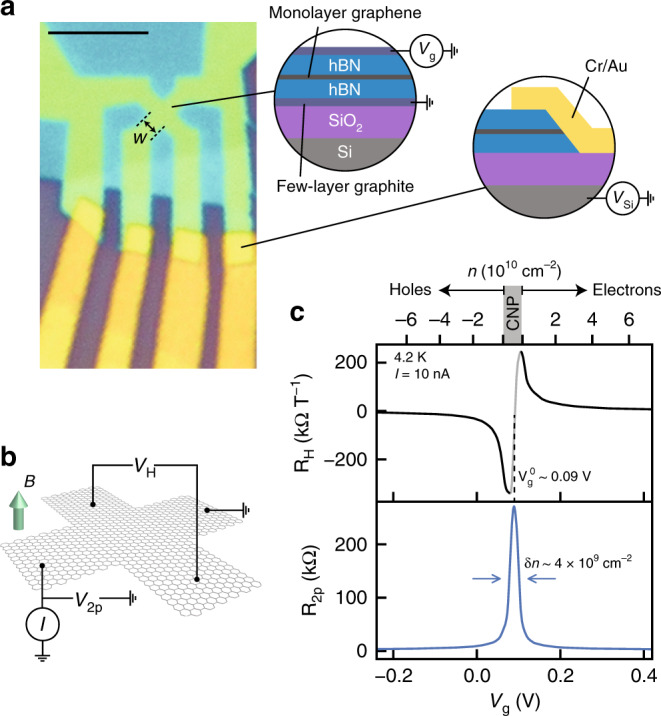
Ultraclean graphene Hall sensors. **a** Optical microscope image of device G1 (*w* = 1 µm, scale bar: 5 µm). Left cross-section: Hall cross layer structure consisting of monolayer graphene encapsulated with hexagonal boron nitride (hBN) and few-layer graphite. Right cross-section: edge contacts. **b** Schematic of the measurement configuration, with Hall voltage *V*_H_, two-point voltage *V*_2p_, bias current *I*, and out-of-plane magnetic field *B*. **c** Top gate voltage (*V*_g_) dependence of the Hall coefficient *R*_H_ and two-point resistance *R*_2p_ at 4.2 K under small ac bias and background fields up to *B* = 100 mT. The upper axis indicates the corresponding electron and hole densities.

Figure 2a shows the structure of our graphite-gated devices along with an optical image of device G1 (see Supplementary Fig. 1 for optical images of additional devices, including metal-gated devices). Each graphite-gated device is fabricated on a silicon substrate from a heterostructure consisting of exfoliated MLG encapsulated with hBN gate dielectrics and FLG gate electrodes assembled using a dry-transfer technique (see Methods). The combination of low charged defect density in hBN and the ability of FLG to screen charged impurity disorder in the silicon substrate improves carrier mobility^20,30^, reduces the charge inhomogeneity^22,23^, and can reduce charge noise in graphene devices^21^. The top gate tunes the carrier density in the active region of the device, while the grounded bottom gate screens the electric field from the silicon back gate. We apply 40 V to the silicon back gate to induce a high electron density in the graphene-based section of the leads. This lowers the resistance of the leads and edge contacts^24^, consequently lowering the voltage noise (see Supplementary Note 1 and Supplementary Fig. 1).

### Hall voltage response

We first evaluate the electronic quality of our devices at low background magnetic field and low temperature in a liquid-helium cryostat. We bias the device with a small ac current *I* and measure the two-point (*V*_2p_) and Hall (*V*_H_) voltages using standard low-frequency lock-in techniques while applying top gate voltage *V*_g_ to tune the carrier density (Fig. 2a, b). From a series of gate sweeps at fixed magnetic field *B* up to 100 mT (see Supplementary Fig. 2), we determine the Hall coefficient *R*_H_ = *I*^−1^(∂*V*_H_/∂*B*)_*B*=0_ and extract the carrier density *n* = 1/(e*R*_H_) (Fig. 2c, upper panel). At gate voltages near the charge neutrality point (CNP), the coexistence of electrons and holes makes the Hall voltage nonlinear in magnetic field^31^. Elsewhere, the Hall voltage is linear in *B* at least up to 100 mT, and *R*_H_ ~ *n*^−1^ ~ *V*_g_^−1^ assuming a simple capacitive coupling of the gate to the mobile carrier density^19^. Extrapolating the electron and hole densities to zero reveals that electrons and holes appear to reach charge neutrality at different *V*_g_. This is consistent with contributions to the charging behavior of the graphene sheet from the quantum capacitance and additional charge traps with nonconstant capacitance, which become significant because of the large gate capacitance and small charge inhomogeneity in our devices^19,32,33^. The maximum (minimum) value of *R*_H_ for electron (hole) doping 240 kΩ T^−1^ (−340 kΩ T^−1^) implies a smallest mobile carrier density *δn* ~ 2.6 × 10^9^ cm^−2^ (−1.8 × 10^9^ cm^−2^) limited by intrinsic charge inhomogeneity. This low charge inhomogeneity is consistent with that reported in other devices with atomically smooth single-crystal graphite gate electrodes^22,23^. The two-point resistance *R*_2p_ = *V*_2p_/*I* (Fig. 2c, lower panel) is sharply peaked, in excess of 200 kΩ at the CNP. The narrow width of this peak implies a charge inhomogeneity ~4 × 10^9^ cm^−2^, in agreement with that obtained using *R*_H_. For moderate electron or hole doping, *R*_2p_ decreases to a few kΩ, with major contributions from the resistance of the graphene channel (~1 kΩ) and edge contacts (~1–2 kΩ).

**Fig. 3:**
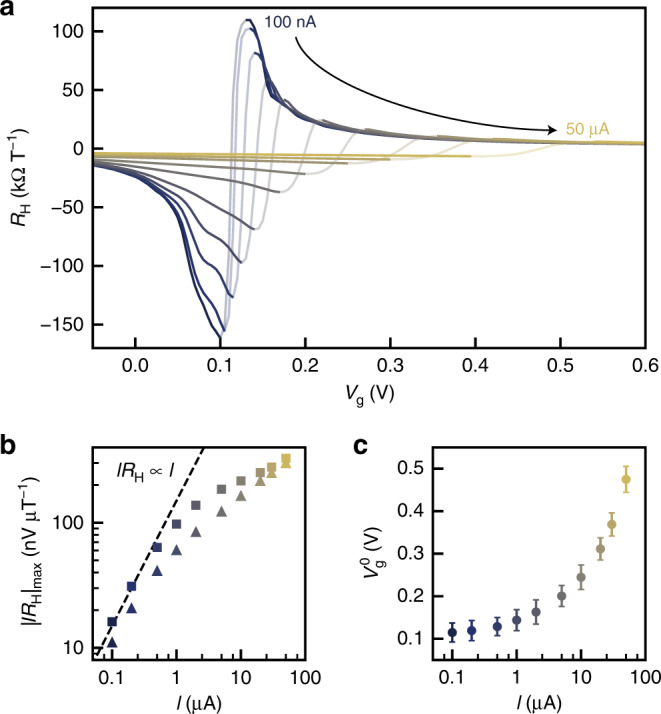
Hall coefficient measurements. **a** Hall coefficient *R*_H_ for device G1 under varying dc current bias at 4.2 K. **b** Bias current dependence of the peak value of *IR*_H_. **c** Bias current dependence of the charge neutrality point voltage *V*_g_^0^. Error bars represent the uncertainty in determining the point at which *R*_H_ crosses zero.

Next, we characterize the voltage response as a function of applied dc current bias up to 50 μA. The Hall voltage response to a small change in magnetic field *δB* is *δV*_H_ = *IR*_H_*δB*, suggesting that applying a larger bias current in principle proportionally increases the voltage signal. In practice, a large dc bias causes two changes in the transport characteristics of the devices (Fig. 3a): the peak *R*_H_ decreases and the CNP gate voltage *V*_g_^0^ shifts. The direction of the shift in *V*_g_^0^ (Fig. 3c) depends on the polarity of the applied current. These changes are consistent with a potential gradient and resulting carrier density gradient across the device^32^ (see Supplementary Note 3 and Supplementary Fig. 3). This modifies the average *R*_H_ within the Hall cross and limits its peak value. Despite the reduction in peak *R*_H_, applying larger bias current still increases the absolute voltage sensitivity *IR*_H_ = (∂*V*_H_/∂*B*)_*B*=0_ (Fig. 3b), giving a larger change in Hall voltage per unit change in magnetic field.

### Voltage noise and detection limit

**Fig. 4:**
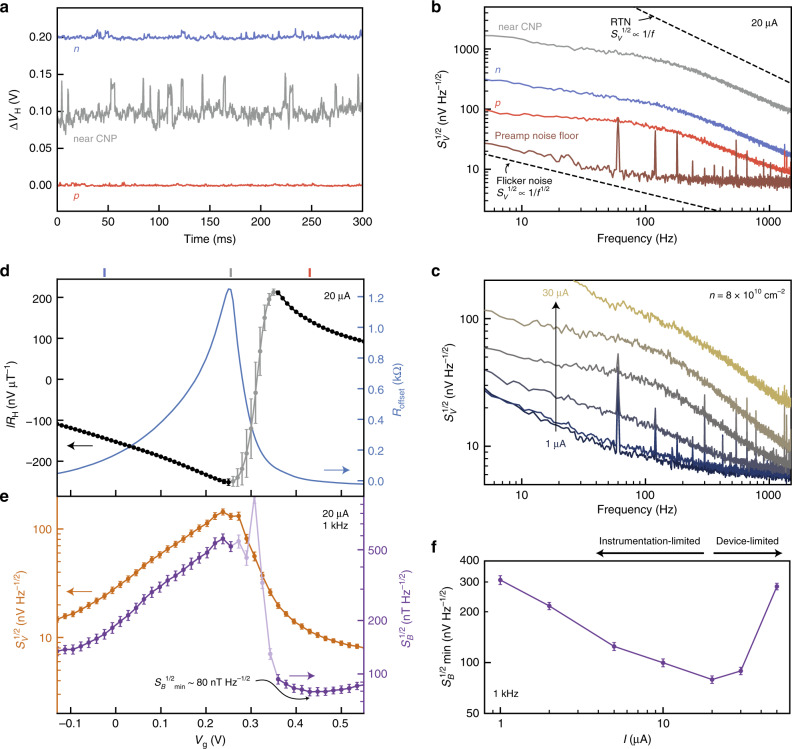
Noise measurements. **a** Time traces of the Hall voltage (offset for clarity) and **b** Hall voltage noise spectral density *S*_*V*_^1/2^ for device G1 at fixed bias current and 4.2 K. The three curves correspond to the gate voltages marked at the top of the upper panel of (**d**). Dashed lines in **b** follow the expected dependence of random telegraph noise (RTN) at high frequency (*f*^−1^) and 1/*f* noise (*f*^−1/2^). **c** Comparison of *S*_*V*_^1/2^ spectra at different bias currents. At each bias current, we set *V*_g_ such that *R*_H_ ≈ 7.8 kΩ T^−1^, corresponding to *n* ≈ 8 × 10^10^ cm^−2^. **d** *IR*_H_ and *R*_offset_ = *V*_H_(*B* = 0)/*I* for 20 μA bias current. **e** *S*_V_^1/2^ and magnetic field detection limit *S*_*B*_^1/2^ at 1 kHz. **f** Bias current dependence of the minimum *S*_*B*_^1/2^ at 1 kHz. In panels **d**–**f**, error bars are determined considering the standard error of the linear fit for *R*_H_ and the standard deviation of *S*_*V*_^1/2^ in a window of width 200 Hz centered at 1 kHz.

To determine the detection limit reported in Fig. 1, we measure the noise performance of the devices alongside the voltage response. We measure fluctuations in the Hall voltage in real time (Fig. 4a) and take a Fourier transform (see Methods) to arrive at the Hall voltage noise spectral density *S*_*V*_^1/2^ (Fig. 4b). At low bias, 60 Hz and preamplifier input noise dominate the *S*_*V*_^1/2^ spectrum (Fig. 4c). The shape of the noise spectrum at higher bias suggests the presence of both flicker noise (1/*f* noise; *S*_*V*_^1/2^ ∝ *f*^−1/2^) and random telegraph noise (RTN; *S*_*V*_^1/2^ constant at low frequency, *S*_*V*_^1/2^ ∝ *f*^−1^ at high frequency), as reported previously in micrometer-scale Hall sensors^11,13,17^ and graphene-based devices^16,21,34^. While 1/*f* noise originates most likely from random charging and discharging events of an ensemble of charge traps, RTN is characteristic of a single charge trap more strongly coupled to the device. These charging events can induce fluctuations in both the carrier mobility and carrier density which are prominent in graphene-based devices at low carrier density^13,16,34^. Charge fluctuations that modulate the contact resistance and defect states in the substrate or etched edges of the device can couple strongly into the voltage noise, especially near charge neutrality where charge fluctuations are poorly screened^16,34^. We find that the behavior of the RTN changes between successive cooldowns and under different conditions of current bias and gate voltage. In Supplementary Note 4, we extract quantitatively the relative contributions of RTN and 1/*f* noise for a typical noise spectrum.

Figure 4e summarizes the low-temperature gate voltage dependence of *S*_*V*_^1/2^ at zero *B* and corresponding magnetic field detection limit *S*_*B*_^1/2^ = *S*_*V*_^1/2^/(*IR*_H_) at 20 μA current bias and 1 kHz. At this frequency, the gate dependence of *S*_*V*_^1/2^ is most apparent; the frequency is low enough that the voltage noise surpasses the instrumentation noise floor, but high enough that the contribution from RTN is small. The shape of the curve in Fig. 4e is similar to that of the offset resistance at zero background magnetic field *R*_offset_ = *V*_H_(*B* = 0)/*I* (Fig. 4d). This offset most likely arises in our case from inhomogeneous current flow at doping levels near charge neutrality and has the effect of coupling in additional 1/*f* noise contributions associated with the longitudinal resistance^11,13^.

Figure 4f shows that a 20 μA bias current minimizes the magnetic field detection limit. At this intermediate bias current, the increase in the voltage signal above the instrumentation noise floor is favorable over the reduction of *R*_H_ at large bias current. Notably, the minimum *S*_B_^1/2^ does not occur at the same value of *V*_g_ at which *R*_H_ peaks. This indicates that the optimum working point of the Hall sensor balances tuning away from the CNP to reduce *S*_*V*_^1/2^ and tuning close to the CNP to increase *R*_H_. The minimum value, *S*_*B*_^1/2^ ~ 80 nT Hz^−1/2^ at 1 kHz (lowermost point in Fig. 1), is to our knowledge the smallest magnetic field detection limit ever reported in a micrometer-scale Hall sensor at 4.2 K. At room temperature, repeating the Hall coefficient and Hall voltage noise measurements (see Supplementary Note 5 and Supplementary Fig. 5c, d) reveals that the detection limit is generally larger, but still competitive with the best Hall sensors reported in the literature (see Fig. 1).

### Performance in large background magnetic field

**Fig. 5:**
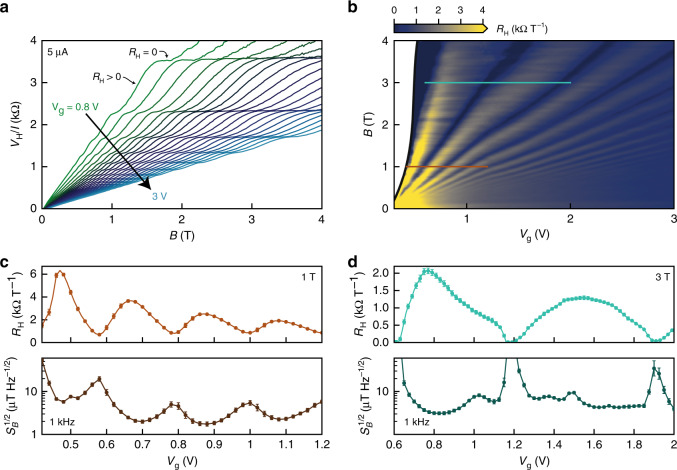
Performance in large background magnetic field. **a** Magnetic field dependence of *V*_H_/*I* in the quantum Hall regime for device G1 at 4.2 K. The curves span gate voltages corresponding to electron density 0.24–1.14 × 10^12^ cm^−2^ at zero field. **b** *R*_H_ determined locally at each point (*V*_g_, *B*). **c**, **d** *R*_H_ and *S*_*B*_^1/2^ at 1 kHz along the horizontal lines in (**b**): **c** *B* = 1 T, **d** *B* = 3 T. Error bars are determined considering the standard error of the linear fit for *R*_H_ and the standard deviation of *S*_*V*_^1/2^ in a window of width 200 Hz centered at 1 kHz. All measurements are performed under 5 μA dc current bias.

Finally, we characterize the detection limit for small changes in magnetic field in the presence of a large magnetic field background. To our knowledge this has not been reported for any high-mobility micrometer-scale Hall sensors. In a large background magnetic field, the Hall resistance develops plateaus (Fig. 5a) spaced by Δ(*V*_H_/*I*)^−1^ = 4e^2^/*h* as expected for MLG in the quantum Hall regime^19^. The deviation of the resistance plateaus from precise quantization is caused by the large bias current and the wide, extended Hall voltage contacts in our device (Fig. 2a), which mix a significant fraction of the longitudinal resistance into the Hall resistance^35^. The Hall coefficient *R*_H_ = *I*^−1^(∂*V*_H_/∂*B*) (Fig. 5b–d) now reaches local minima at values of (*B*, *V*_g_) corresponding to the resistance plateaus. At high magnetic field, the resistance plateaus flatten (*R*_H_ = 0). Repeating measurements of the Hall voltage noise as described above, at 3 T we obtain *S*_*B*_^1/2^ ~ 3 μT Hz^−1/2^ at optimum carrier density tuning (Fig. 5d, *V*_g_ ~ 0.8 V). The larger detection limit compared to measurements at zero field is a result of both the reduced *R*_H_ and a general increase in voltage noise in large background magnetic field, which is correlated with large longitudinal magnetoresistance and may also be attributed to charge fluctuations between localized and extended quantum Hall states^36,37^.

## Discussion

In summary, we show that hBN-encapsulated MLG combined with FLG gates is an excellent material system for micrometer-scale Hall sensors. Currently, the only way to obtain ultraclean graphene devices is to fabricate them individually from exfoliated layers, resulting in devices on the few-micrometer scale. However, our work directly provides information and motivation for the development of material growth for bulk fabrication of ultraclean graphene sensors.

It is insightful to compare the performance of our Hall sensors to SQUID magnetometers, which are among the most sensitive magnetic field sensors available^5^. For planar niobium-based SQUIDs, a typical magnetic flux detection limit *S*_Φ_^1/2^ is ~1 μΦ_0_ Hz^−1/2^ at kHz frequencies, where Φ_0_ is the magnetic flux quantum, independent of the area *A* of the sensitive region^8,9^. For a homogenous magnetic field across the sensitive area, the corresponding magnetic field detection limit for a SQUID scales with 1/*A*. In the case of *S*_Φ_^1/2^ ~ 1 μΦ_0_ Hz^−1/2^, *S*_*B*_^1/2^ ~ 40 nT Hz^−1/2^ for a circular sensitive area with a 0.25 μm diameter or *S*_*B*_^1/2^ ~ 3 nT Hz^−1/2^ for a 1 μm diameter. While this is superior to the detection limit of the Hall sensors reported here, it is still comparable. Given that the noise performance is still limited by instrumentation for our Hall sensors, they have the potential to outperform sub-micron SQUIDs^9^ following implementation of more sophisticated read-out techniques.

Moreover, the Hall sensors reported here work in a much less restricted parameter space than SQUIDs. Importantly, by tuning the carrier density we demonstrate optimization of the detection limit over a large range of both temperature and magnetic field. The dry-transfer fabrication process offers flexibility to fabricate Hall sensors directly on top of materials of interest or incorporate the devices into a scanning probe. Scanning Hall probe microscopy with a high-performing graphene sensor will enable the imaging of magnetic fields over a combined range of temperatures and magnetic fields not accessed with a single scanning probe to date. The detection of small magnetic field variations with a single solid-state sensor over a broad parameter space is promising for studying a range of condensed matter systems including unconventional superconductors across their magnetic field-temperature phase diagram, magnetic-field-tuned phases of matter, and electric currents in regimes of electronic transport that appear at high temperature and magnetic field.

## Methods

### Device fabrication

We obtain MLG, FLG, and ~20–40 nm thick hBN flakes via mechanical exfoliation of bulk Kish graphite (Graphene Supermarket or CoorsTek) and hBN crystals grown using a high-pressure technique^38^. We repeatedly cleave the bulk crystals using Scotch Magic tape and press the tape onto degenerately doped silicon wafers with 285 nm SiO_2_ (Nova Electronic Materials) treated with a gentle oxygen plasma. To increase the yield of large-area flakes, we heat for 5 min at 100 °C, and let the chips return to room temperature before removing the tape^39^. We identify suitable flakes for devices only using optical inspection.

We create heterostructures with layer structure hBN/FLG/hBN/MLG/hBN/FLG/SiO_2_/Si (G1–G3) or hBN/MLG/hBN/SiO_2_/Si (M1 and M2) using a dry-transfer technique^20,40^. The transfer slide consists of a thin sheet of poly(bisphenol A carbonate) (PC, Sigma Aldrich 435139) on top of a PDMS stamp (Gel-Pak) with curved top surface^41^, allowing for precise control over the engagement of the stamp onto the substrate. The top hBN (~5 nm) only facilitates pickup of the other flakes and does not in principle influence the electronic properties of the device. We pick up flakes sequentially at 80 °C and heat the final silicon substrate at 180 °C before releasing the stack, ensuring that bubbles trapped between the flakes are pushed towards the edges of the stack upon engaging^42,43^. We intentionally misalign the straight edges of the graphene and hBN flakes by ~15° to avoid creating a Moiré pattern between the graphene and hBN sheets^24^. Finally, we dissolve the PC in chloroform for ~4 h, rinse with isopropyl alcohol, and blow dry with nitrogen. A final anneal in high vacuum (<10^−6^ Torr) for 3 h at 300 °C is effective in removing polymer residues from the transfer.

We employ standard nanofabrication techniques to etch the devices into Hall crosses, expose a one-dimensional graphene edge^20^, and make edge contacts (3 nm Cr/40 nm Pd/40 nm Au or 3 nm Cr/80 nm Au) to the MLG and FLG layers. For devices M1 and M2, we evaporate 5 nm Ti/30 nm Au/10 nm Pt directly onto the top hBN and use the metal top gate as part of the etch mask. Importantly, we have developed process conditions that help reduce the contact resistance. We use a CHF_3_/O_2_/Ar inductively coupled plasma (20/10/10 sccm, 10 mTorr, 30 W ICP, 10 W RF) selective towards etching hBN. Previous work suggests that selective etching reduces the contact resistance by increasing the metal–graphene contact area^44^. Finally, we emphasize that to achieve consistently working contacts, we found it necessary to use an electron-beam evaporator with low base pressure (~10^−7^ Torr) and a rotating sample chuck.

### DC transport and noise measurements

The same wiring and instrumentation are used for both Hall voltage and noise measurements under dc current bias. We apply dc current using a constant-current source (Keithley 2400) and a series ~1 MΩ bias resistor. We amplify and filter the Hall voltage using a preamplifier (Signal Recovery 5113, 10 kHz lowpass filter) and obtain time traces using the input terminal of a lock-in amplifier (Zurich Instruments MFLI). The preamplifier is in dc coupling mode for Hall voltage measurements and in ac coupling mode (with larger gain) for noise measurements. In the latter case, we record 30 time traces sampled at 3.66 kHz for ~4.5 s each, giving 2^14^ sampled points per time trace. The Fourier transform of each time trace is computed using Welch’s method^45^ with a Hann window. We use frequency bins with 50% overlap consisting of 27 points to reduce variance. The resulting power spectral density *S*_*V*_ is valid in a frequency band spanning ~1 Hz to ~3.66 kHz. For the purpose of comparing the noise under different experimental conditions, we take a root-mean-square average over a 200 Hz band centered at 1 kHz and report the uncertainty as the standard deviation of the averaged samples.

## Supplementary information

Supplementary Information

## Data Availability

The data that support the findings of this study are available from the corresponding author upon request.
