# Publisher Correction: Magnetic field detection limits for ultraclean graphene Hall sensors

**DOI:** 10.1038/s41467-021-20969-z

**Published:** 2021-01-18

**Authors:** Brian T. Schaefer, Lei Wang, Alexander Jarjour, Kenji Watanabe, Takashi Taniguchi, Paul L. McEuen, Katja C. Nowack

**Affiliations:** 1 Laboratory of Atomic and Solid State Physics, Cornell University, Ithaca, NY 14853 USA; 2 Kavli Institute at Cornell for Nanoscale Science, Cornell University, Ithaca, NY 14853 USA; 3 National Institute for Materials Science, 1-1 Namiki, Tsukuba, 305-0044 Japan

**Keywords:** Electronic properties and devices, Sensors, Electronic and spintronic devices

Correction to: *Nature Communications* 10.1038/s41467-020-18007-5, published online 20 August 2020.

The original version of this Article contained an error in Fig. 4f, in which the units on the vertical axis should be “(nT Hz^−1/2^)”, as opposed to “(nV Hz^−1/2^)”.

This has been corrected in both the PDF and HTML versions of the Article.

