# Supplementary material for: Magnetic field detection limits for ultraclean graphene Hall sensors
Source: Nat Commun. 2020 Aug 20;11:4163. doi: 10.1038/s41467-020-18007-5 (PMC7441171; doi:10.1038/s41467-020-18007-5)
Supplement: Supplementary file 1 — Supplementary Information [file 41467_2020_18007_MOESM1_ESM.pdf]

Supplementary Information

Schaefer et al.

Magnetic field detection limits for ultraclean graphene Hall sensors

Brian T. Schaefer,[1] Lei Wang,[2] Alexander Jarjour,[1] Kenji Watanabe,[3]
Takashi Taniguchi,[3] Paul L. McEuen,[1,2] and Katja C. Nowack[1,2]

[1]*Laboratory of Atomic and Solid-State Physics, Cornell University, Ithaca, NY 14853, USA*

[2]*Kavli Institute at Cornell for Nanoscale Science, Cornell University, Ithaca, NY 14853, USA*

[3]*National Institute for Materials Science, 1-1 Namiki, Tsukuba 305-0044, Japan*

Material	w (μm)	$S_B^{1/2}$ (nT Hz$^{-1/2}$)	$S_B^{1/2}w$ (μm nT Hz$^{-1/2}$)	Temperature
G/SiC[a] [2]	0.5	(79500, 101000)	(40000, 50500)	300 K
	5	(6300, 8000)	(31500, 40000)	300 K
	10	(4000, 5100)	(40300, 51000)	300 K
G[b] [3]	1.5	10000	15000	300 K
	1.5	4000	6000	4 K
G[a] [6]	0.085	(40400, 46000)	(3400, 3900)	300 K
	1	(4100, 4700)	(4100, 4700)	300 K
hBN/G/hBN [10]	3	175	(525, 875)	300 K
GaAs [4]	0.8	25000	20000	300 K
	2	6000	12000	300 K
	10	1700	17000	300 K
	20	700	14000	300 K
	40	200	8000	300 K
GaAs[a] [9]	0.35	(1300, 2000)	(440, 700)	4 K
InGaAs [8]	1.5	800	1200	300 K
InSb [5]	0.6	12000	7200	300 K
InSb [7]	1.5	600	800	300 K
InAsSb[c] [11]	–	58	–	300 K
Si [4]	5	1000	5000	300 K
	10	1000	10000	300 K
	20	470	9400	300 K
Bi [1]	0.05	80000	4000	300 K

Supplementary Table 1. Lateral size w and magnetic field detection limit $S_B^{1/2}$ at 1 kHz extracted from the literature, used in Figure 1 in the main text. Entries expressed as a pair of numbers are estimates of lower and upper bounds (see footnotes).

[a] $S_B^{1/2}$ extrapolated to 1 kHz assuming $1/f$ noise scales as $f^{-\alpha}$ $(0.4 < \alpha < 0.6)$.
[b] Width inferred from optical image.
[c] Ref. [11] does not clearly state the size of the device for which the detection limit is reported, and we do not include this work in Figure 1 in the main text. Although the authors show an image of a device with $w = 1$ μm, we estimate from the reported carrier density and carrier mobility that this device would have series resistance > 14 kΩ, a factor of 10 larger than the resistance stated for the device exhibiting the reported detection limit.

Supplementary Note 1. Performance of additional devices

Supplementary Table 2 and Supplementary Figure 1 describe the devices fabricated for this study. The device structure either consists of top and bottom few-layer graphite gates (G1, G2, G3, Supplementary Figure 1b inset) or a single top metal gate (M1, M2, Supplementary Figure 1c inset). In most devices, the charge inhomogeneity δn is comparable to that reported previously in hBN-encapsulated graphene devices [12, 13]. We speculate that poorly screened charge disorder from the etched device edges increases the effective δn for G2 and G3 as compared to G1 [14, 15]. Assuming that electrons and holes each contribute $\delta n/2$ to the total charge inhomogeneity, the Fermi wavelength is $\lambda_{\mathrm{F}} = 2\pi/\sqrt{\pi(\delta n/2)}$. If λ_{F} is bounded by the device size $w = 1$ µm, we can estimate $\delta n \sim 10^{10}$ cm^{-2} for G2 and G3 in agreement with our measurements.

The effective δn (indicated by the maximum Hall coefficient $R_{\mathrm{H}}^{\mathrm{max}}$) depends on both gating and bias conditions. As we discuss in the main text, increasing the bias current decreases $R_{\mathrm{H}}^{\mathrm{max}}$ by effectively increasing δn (see Supplementary Note 3). This effect is similar for both graphite-gated and metal-gated devices (Supplementary Figure 1f), but graphite-gated devices with lower intrinsic δn can still retain a larger $R_{\mathrm{H}}^{\mathrm{max}}$ at high bias current. Other than leading to lower δn, the bottom graphite gate also enables independent doping of the contacts and Hall cross. In device M1 lacking a bottom graphite gate, the silicon gate decreases $R_{\mathrm{H}}^{\mathrm{max}}$ (Supplementary Figure 1g). However, in device G1, doping the contacts reduces both the two-point resistance and voltage noise (Supplementary Figure 1h) without decreasing $R_{\mathrm{H}}^{\mathrm{max}}$.

		w (µm)	δn (cm^{-2})	${S_B^{1/2}}_{\mathrm{min}}$ (nT Hz$^{-1/2}$)
Graphite-gated devices	G1	1	$\sim 4 \times 10^9$	80
	G2	0.5	$\sim 10^{10}$	150
	G3	0.5	$\sim 10^{10}$	200
Metal-gated devices	M1	1	$\sim 10^{10}$	250
	M2	2	$\sim 10^{10}$	100

Supplementary Table 2. Summary of the performance of additional devices at 4.2 K. δn is estimated from the width of the peak in the two-point resistance.

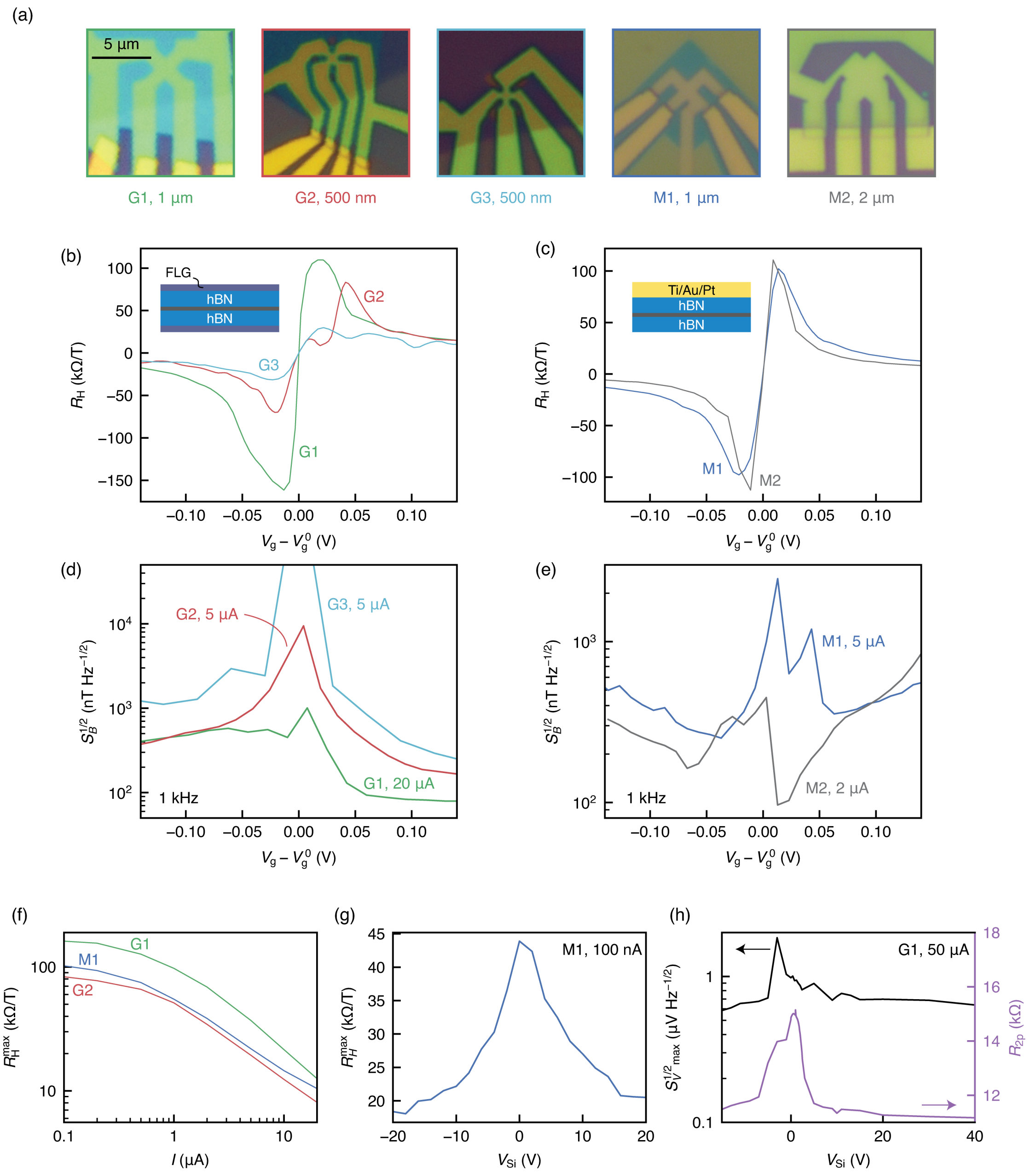

Supplementary Figure 1. **Characterization of additional devices.** **a** Optical images of the devices summarized in Table 2. **b-c** Hall coefficient (R_H) measurements of devices under 100 nA DC bias at 4.2 K. The insets illustrate the layer structure of the devices represented in each panel. **d-e** Magnetic field detection limit $S_B^{1/2}$ at 1 kHz and 4.2 K, shown for the current that yields the minimum $S_B^{1/2}$ for each device. We report measurements for both graphite-gated (**b**, **d**) and metal-gated (**c**, **e**) devices. **f** Current bias dependence of peak R_H. **g** Reduction in peak R_H upon applying voltage to the silicon gate of M1. **h** Reduction of dc two-point resistance and peak voltage noise at 1 kHz upon applying silicon gate voltage to G1.

Supplementary Note 2. Quantum hall resistance plateaus at low bias current and magnetic field

The appearance of well-defined quantum Hall resistance plateaus at low magnetic fields is a clear signature of small charge inhomogeneity in high-quality graphene devices [16]. In most of our measurements, we consider the regime of large bias current to increase the Hall voltage and do not observe resistance plateaus developing until ∼500 mT (Fig. 5a). At low bias current, the exceptionally small charge inhomogeneity is evident through the appearance of quantum Hall resistance plateaus developiung at magnetic field as low as ∼40 mT at liquid-helium temperature (Supplementary Figure 2).

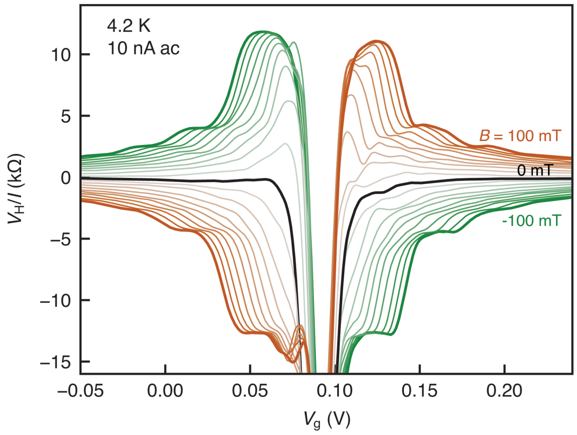

Supplementary Figure 2. AC Hall response at low magnetic field. Hall resistance V_{H}/I of device G1 measured versus gate voltage V_{g} and a series of magnetic fields B in steps of 10 mT.

Supplementary Note 3. Carrier density gradient under large current bias

Applying a large bias current to our devices strongly modifies the relationship between Hall coefficient and gate voltage. Here, we show that our measurements are consistent with carrier density gradients resulting from the large bias current.

We consider an $L \times L$ square device with contacts spanning the entire length of each of the four edges (Supplementary Figure 3a) that measure the average Hall voltage in the center square. The top and bottom contacts are the Hall voltage leads, the device is biased with constant current I from the left contact (potential $\psi(x = 0) = IR_{2\mathrm{p}}$), and the right contact is grounded ($\psi(x = L) = 0$).

The electron (n_g) and hole (p_g) densities away from the CNP depend on the potential difference between the gate and the graphene layer:

$$n_g(x) = \frac{C_g}{e}[V_g - \psi(x)] \qquad p_g(x) = \frac{C_g}{e}[\psi(x) - V_g],$$

where C_g is the gate capacitance. Accounting for charge inhomogeneity δn near the Dirac point, the electron and hole densities become [17]

$$n(x) = \frac{n_g + \sqrt{n_g^2 + \delta n^2}}{2} \qquad p(x) = \frac{p_g + \sqrt{p_g^2 + \delta n^2}}{2}.$$

Noting $n_g^2 = p_g^2$ and $n_g + p_g = 0$, the total carrier density is:

$$n(x) + p(x) = \sqrt{n_g^2 + \delta n^2} = \sqrt{\frac{C_g^2}{e^2}[V_g - \psi(x)]^2 + \delta n^2}.$$

Finally, using the resistivity $\rho^{-1} = e\mu(n + p)$, the Ohmic potential drop is given by:

$$\frac{\partial \psi}{\partial x} = -\frac{I\rho(x)}{L} = -\frac{I}{Le\mu[n(x) + p(x)]} = -\frac{I}{Le\mu\sqrt{\frac{C_g^2}{e^2}[V_g - \psi(x)]^2 + \delta n^2}}.$$

Solving this differential equation numerically with initial condition $\psi(L) = 0$ reveals that the potential $\psi(x)$ drops nonlinearly along the device channel (Supplementary Figure 3b). We extract the electron and hole densities $n(x)$ and $p(x)$ (Supplementary Figure 3e), two-point resistance $R_{2\mathrm{p}} = \psi(0)/I$, and average Hall coefficient R_H using a two-carrier magnetoresist-

ance model and average electron and hole densities in the channel [18]:

$$R_H = \frac{1}{e}\frac{\bar{n}-\bar{p}}{(\bar{n}+\bar{p})^2}.$$

Our calculation (Supplementary Figure 3d) demonstrates many qualitative similarities to our measurements (Supplementary Figure 3c), namely electron-hole asymmetry, a broadened Dirac peak, and a reduced peak Hall coefficient. Increasing the charge inhomogeneity (Supplementary Figure 3f) or bias current (Supplementary Figure 3g) further reduces the peak Hall coefficient, consistent with our measurements.

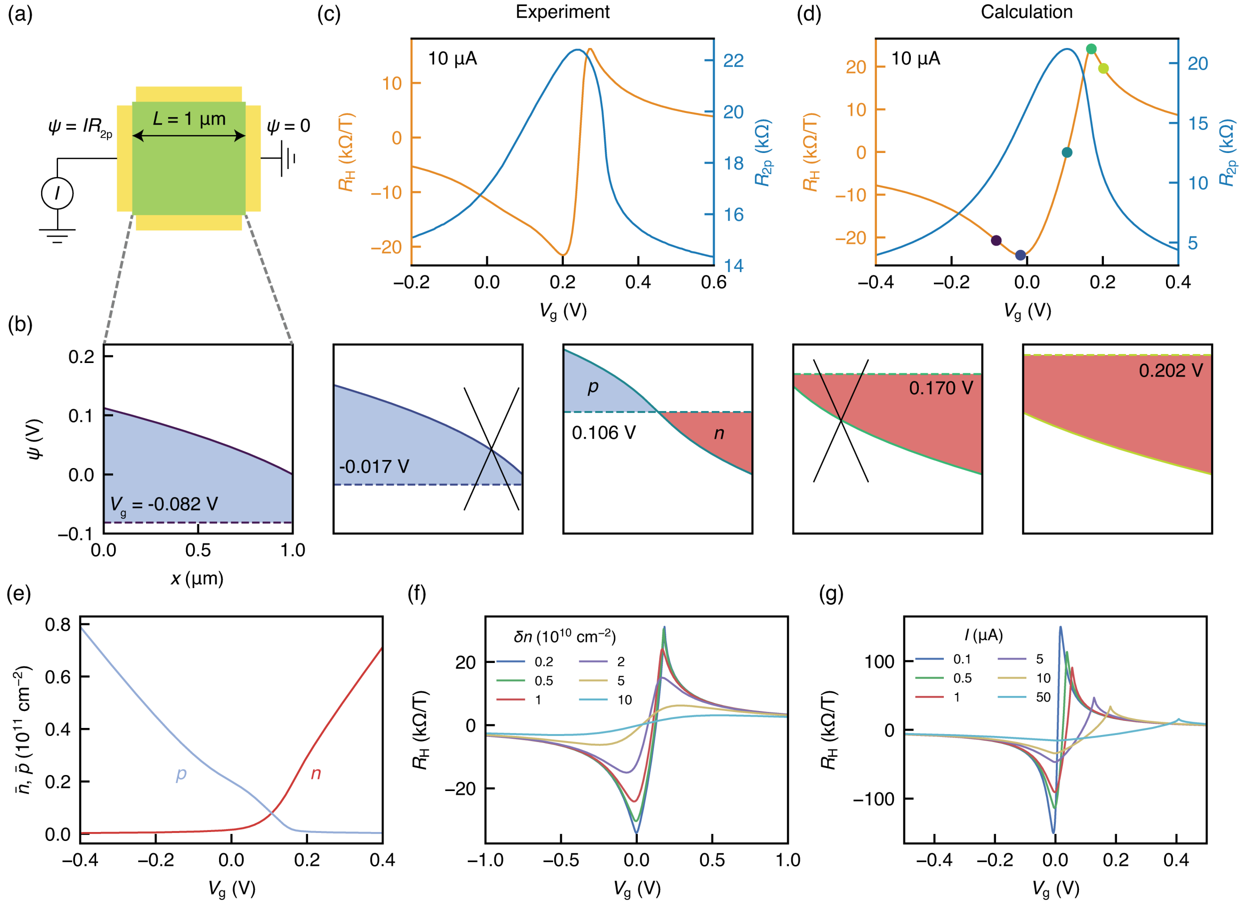

Supplementary Figure 3. Carrier density gradient under large DC current bias. **a** Schematic of the model device. **b** Potential profiles across the Hall cross corresponding to the markers in (**d**). Dashed lines indicate the position of V_g, and the shading represents the carrier density (illustrated by Dirac cones in two of the panels). **c,d** Measured (**c**) and calculated (**d**) R_H and R_2p under 10 µA dc bias current. The calculation uses $\mu = 20000\ \mathrm{cm}^2\ \mathrm{V}^{-1}\ \mathrm{s}^{-1}$, $C_g = 0.03\ \mathrm{\mu F\,cm}^{-2}$, and $\delta n = 10^{10}\ \mathrm{cm}^{-2}$. **e** Calculated average electron and hole densities in the Hall cross. **f,g** Calculated charge inhomogeneity (**f**) and bias current (**g**) dependence of R_H.

Supplementary Note 4. Contributions of noise sources to the detection limit

Johnson noise

In our devices, the white Johnson noise is always smaller than the charge noise contriubtions from $1/f$ and random telegraph noise. The Johnson noise spectral density $S_V^{1/2} = \sqrt{4k_BTR}$ is at most ∼10 nV $\mathrm{Hz}^{-1/2}$ for a maximum $R_{2\mathrm{p}}$ of ∼250 kΩ at liquid-helium temperature (main text) or ∼18 nV $\mathrm{Hz}^{-1/2}$ for ∼20 kΩ at room temperature (Supplementary Figure 5c). Ref. [24] notes that because Johnson noise is independent of sensor size, the corresponding magnetic field detection limit $S_B^{1/2} \propto w^{-1}$, similarly to $1/f$ noise.

Random telegraph noise

Although the general behavior of our devices remains the same between cooldowns, the specific amplitude of RTN and gate voltage region over which it is significant tend to change. To illustrate this, we present noise measurements taken during two successive cooldowns, one in which RTN is only present for a small range of gate voltages and another in which RTN is almost completely absent. These measurements are performed in the same way as in the main text, but the wiring used for these measurements involves twisted pairs which add a parasitic capacitance to ground that may suppress the noise slightly at frequencies approaching 1 kHz.

In Cooldown B (Supplementary Figure 4e, lower panel), the nearly linear noise spectra are clearly dominated by $1/f$-like noise, with a slight curvature due to weak RTN. However, in Cooldown A (Supplementary Figure 4e, upper panel), the noise spectra flatten below ∼30 Hz and fall off as f^{-1} at high frequency, characteristic of a Lorentzian RTN spectrum [25]. In the time domain, the voltage flucutates mainly between two distinct voltage states (Supplementary Figure 4a,b). The distribution of voltages comprising each of the two states is Gaussian (Supplementary Figure 4c), while the lifetimes t_1 and t_2 each follow a Poisson distribution (Supplementary Figure 4d) [26]. Fitting the lifetimes to an $\exp(-t/\tau)$ dependence yields a mean lifetime of $\tau_1 = 3.9$ ms for the upper state and $\tau_2 = 49$ ms for the lower state.

The total voltage noise spectral density can be modeled using [25]

$$S_V = \frac{4\delta V^2}{\tau_1 + \tau_2} \frac{\tau^2}{1 + (2\pi f \tau)^2} + \frac{A}{f^\alpha}, \tag{1}$$

where f is the frequency, $\tau^{-1} = \tau_1^{-1} + \tau_2^{-1}$, A is the flicker noise amplitude, and $\alpha \sim 1$. We fit the uppermost spectrum in Supplementary Figure 4e (black curve) fixing $\alpha = 1$ and obtain best-fit parameters $\delta V = 52.5 \pm 0.5$ µV, $\tau_1 = 6.09 \pm 0.09$ ms, $\tau_2 = 49.0 \pm 0.9$ ms, and $A = (3.1 \pm 0.3) \times 10^{-12}$ V.

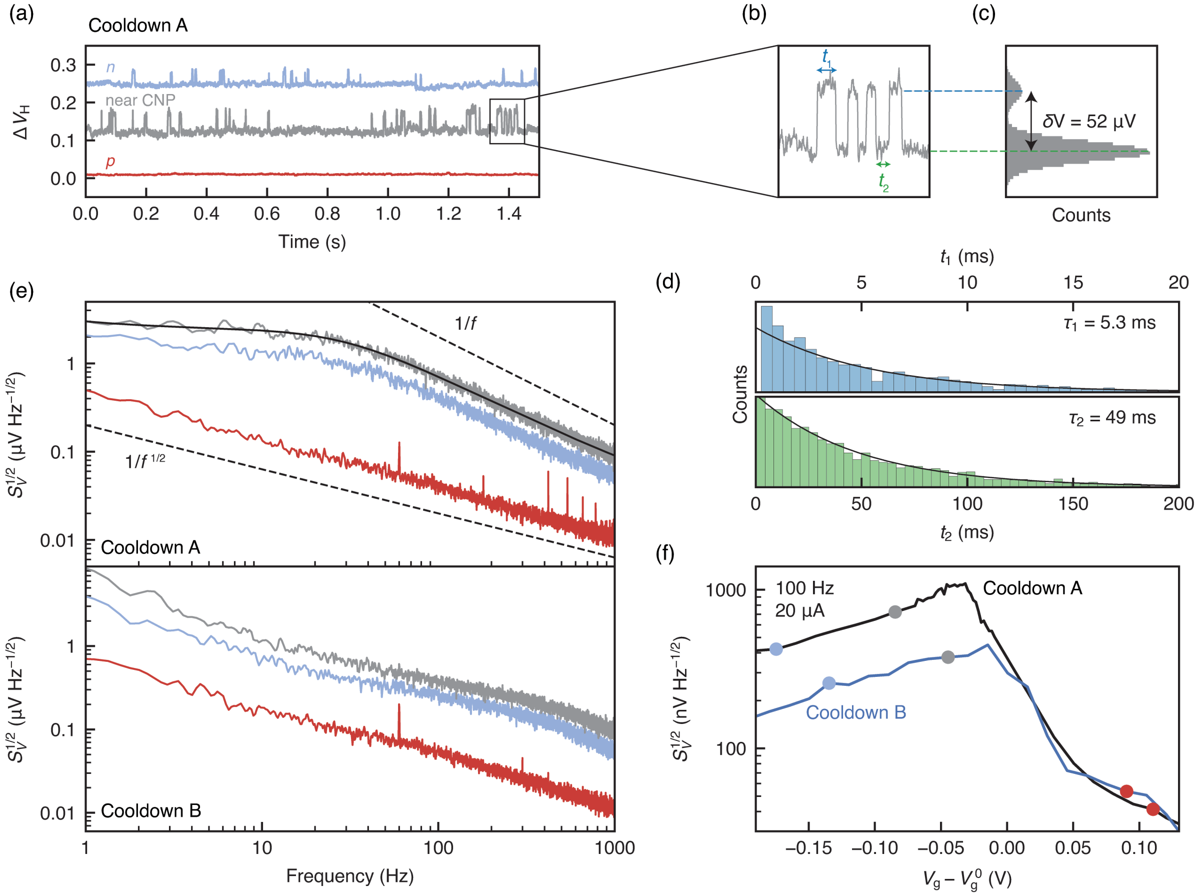

Supplementary Figure 4. Random telegraph noise. **a** Hall voltage time traces at three different gate voltages, measured for device G1 during Cooldown A. Gate voltages correspond to the spectra in **e**. **b** Zoom-in of a voltage trace fluctuating between two voltage states with lifetimes t_1 and t_2. **c** Voltage histogram from the entire 2.2-second time trace. **d** Histograms of the lifetimes of the two voltage states. **e** $S_V^{1/2}$ spectra measured at 1 kHz. The solid curve is a fit to Equation 1. Both sets of spectra were acquired on device G1, but during separate cooldowns. Spectra correspond to the markers in **f**. **f** Average $S_V^{1/2}$ at 100 Hz.

Supplementary Note 5. Temperature dependence of hall coefficient and noise measurements at room temperature

Using a Quantum Design Physical Property Measurement System, we measure R_{H} as a function of gate voltage and temperature (Supplementary Figure 5a). To save time, we estimate R_{H} using measurements only at ±50 mT. Extracting the peak R_{H} at each temperature (Supplementary Figure 5b), we observe that $R_{\mathrm{H}}^{\mathrm{max}}$ shows weak temperature dependence at low temperature and decreases as T^{-2} at high temperature. Modeling the potential fluctuations due to charge disorder as a Gaussian distribution with amplitude Δ, the charge inhomogeneity at the Dirac point is approximately [27]

$$\delta n(T) = \frac{1}{2\pi(\hbar v_{\mathrm{F}})^2}\left[\Delta^2 + \frac{\pi^2}{3}(k_B T)^2\right],$$

where $\hbar$ is the reduced Planck constant, $v_{\mathrm{F}} = 10^6$ m/s is the Fermi velocity, and $k_B T$ is the thermal energy. In Supplementary Figure 5b, we plot $(\delta n(T)e)^{-1}$ for $\Delta = 9$ meV (closely matching the 10 nA data) and $\Delta = 32$ meV (closely matching the 20 µA data). For small bias, the crossover into the T^{-2} regime occurs at a lower temperature than predicted by the model, likely due to reduction of R_H via thermal activation of holes [28].

At room temperature (∼300 K), we perform full characterization of device G1 using the same cryostat insert used for low-temperature measurements, instead positioned between the poles of a C-frame electromagnet (GMW Associates, model 5403). Notably, the bias current has little effect on R_H below ∼20 µA because the thermal charge inhomogeneity exceeds the additional effective inhomogeneity from the bias current (Supplementary Figure 5c). Supplementary Figure 5d illustrates that $S_V^{1/2}$ and $S_B^{1/2}$ have a similar dependence on gate voltage as at low temperature, reaching a minimum $S_B^{1/2} \sim 700$ nT Hz$^{-1/2}$ for small hole doping.

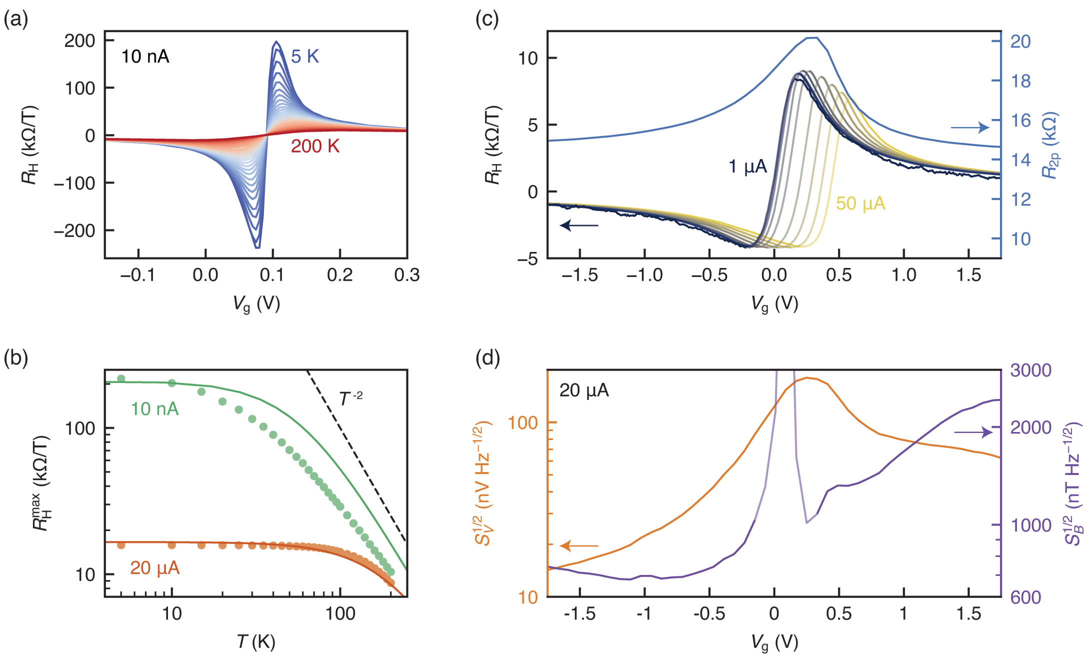

Supplementary Figure 5. **Influence of temperature on Hall coefficient and detection limit.** **a** R_{H} measured as a function of temperature. **b** Temperature dependence of peak R_{H} (markers) and comparison to the theoretical temperature dependence of charge inhomogeneity (solid curves). **c** R_{H} and $R_{2\mathrm{p}}$ at room temperature for 20 μA bias current. **d** $S_{\mathrm{V}}^{1/2}$ and $S_{\mathrm{B}}^{1/2}$ at room temperature. All measurements are performed on device G1.

[1] Sandhu, A., Kurosawa, K., Dede, M. & Oral, A. 50 nm Hall Sensors for Room Temperature Scanning Hall Probe Microscopy. *Jpn. J. Appl. Phys* **43**, 777–778 (2004).

[2] Panchal, V. *et al.* Small epitaxial graphene devices for magnetosensing applications. *J. Appl. Phys.* **111**, 07E509 (2012).

[3] Sonusen, S., Karci, O., Dede, M., Aksoy, S. & Oral, A. Single layer graphene Hall sensors for scanning Hall probe microscopy (SHPM) in 3–300K temperature range. *Appl. Surf. Sci.* **308**, 414–418 (2014).

[4] Vervaeke, K., Simoen, E., Borghs, G. & Moshchalkov, V. V. Size dependence of microscopic Hall sensor detection limits. *Rev. Sci. Instrum.* **80**, 074701 (2009).

[5] Kazakova, O. *et al.* Ultrasmall particle detection using a submicron Hall sensor. *J. Appl. Phys.* **107**, 09E708 (2010).

[6] Collomb, D., Li, P. & Bending, S. J. Nanoscale graphene Hall sensors for high-resolution ambient magnetic imaging. *Sci. Rep.* **9**, 14424 (2019).

[7] Oral, A. *et al.* Room-temperature scanning Hall probe microscope (RT-SHPM) imaging of garnet films using new high-performance InSb sensors. *IEEE Trans. Magn.* **38**, 2438–2440 (2002).

[8] Chenaud, B. *et al.* Sensitivity and noise of micro-Hall magnetic sensors based on InGaAs quantum wells. *J. Appl. Phys.* **119**, 024501 (2016).

[9] Hicks, C. W., Luan, L., Moler, K. A., Zeldov, E. & Shtrikman, H. Noise characteristics of 100 nm scale GaAs/$Al_xGa_{1-x}As$ scanning Hall probes. *Appl. Phys. Lett.* **90**, 133512 (2007).

[10] Dauber, J. *et al.* Ultra-sensitive Hall sensors based on graphene encapsulated in hexagonal boron nitride. *Appl. Phys. Lett.* **106**, 193501 (2015).

[11] Bando, M. *et al.* High sensitivity and multifunctional micro-Hall sensors fabricated using InAlSb/InAsSb/InAlSb heterostructures. *J. Appl. Phys.* **105**, 07E909 (2009).

[12] Wang, L. *et al.* One-Dimensional Electrical Contact to a Two-Dimensional Material. *Science* **342**, 614–617 (2013).

[13] Kretinin, A. V. *et al.* Electronic Properties of Graphene Encapsulated with Different Two-Dimensional Atomic Crystals. *Nano Lett.* **14**, 3270–3276 (2014).

[14] Das Sarma, S., Adam, S., Hwang, E. H. & Rossi, E. Electronic transport in two-dimensional

graphene. *Rev. Mod. Phys.* **83**, 407–470 (2011).

[15] Halbertal, D. *et al.* Imaging resonant dissipation from individual atomic defects in graphene. *Science* **358**, 1303–1306 (2017).

[16] Zomer, P. J., Guimarães, M. H. D., Brant, J. C., Tombros, N. & van Wees, B. J. Fast pick up technique for high quality heterostructures of bilayer graphene and hexagonal boron nitride. *Appl. Phys. Lett.* **105**, 013101 (2014).

[17] Dorgan, V. E., Bae, M.-H. & Pop, E. Mobility and saturation velocity in graphene on SiO2. *Appl. Phys. Lett.* **97**, 082112 (2010).

[18] Wehrfritz, P. & Seyller, T. The Hall coefficient: a tool for characterizing graphene field effect transistors. *2D Mater.* **1**, 035004 (2014).

[19] Balandin, A. A. Low-frequency $1/f$ noise in graphene devices. *Nat. Nanotechnol.* **8**, 549–555 (2013).

[20] Hooge, F. N., Kleinpenning, T. G. M. & Vandamme, L. K. J. Experimental studies on 1/f noise. *Rep. Prog. Phys.* **44**, 479 (1981).

[21] Kleinpenning, T. G. M. Theory of noise investigations on conductors with the four-probe method. *J. Appl. Phys.* **48**, 2946 (1977).

[22] Geim, A. K. *et al.* Ballistic Hall micromagnetometry. *Appl. Phys. Lett.* **71**, 2379 (1997).

[23] Peeters, F. M. & Li, X. Q. Hall magnetometer in the ballistic regime. *Appl. Phys. Lett.* **72**, 572 (1998).

[24] Kirtley, J. R. Fundamental studies of superconductors using scanning magnetic imaging. *Rep. Prog. Phys.* **73**, 126501 (2010).

[25] Machlup, S. Noise in Semiconductors: Spectrum of a Two-Parameter Random Signal. *J. Appl. Phys.* **25**, 341–343 (1954).

[26] Yuzhelevski, Y., Yuzhelevski, M. & Jung, G. Random telegraph noise analysis in time domain. *Rev. Sci. Instrum.* **71**, 1681–1688 (2000).

[27] Li, Q., Hwang, E. H. & Sarma, S. D. Disorder-induced temperature-dependent transport in graphene: Puddles, impurities, activation, and diffusion. *Phys. Rev. B* **84**, 115442 (2011).

[28] Zhu, W., Perebeinos, V., Freitag, M. & Avouris, P. Carrier scattering, mobilities, and electrostatic potential in monolayer, bilayer, and trilayer graphene. *Phys. Rev. B* **80**, 235402 (2009).
